# Clinical outcome and healing rate after meniscal bucket handle tear repair

**DOI:** 10.1186/s12891-022-06037-7

**Published:** 2022-12-05

**Authors:** Lukas N. Muench, Andrea Achtnich, Lukas Krivec, Theresa Diermeier, Klaus Woertler, Sepp Braun, Andreas B. Imhoff, Lukas Willinger

**Affiliations:** 1 Department of Orthopaedic Sports Medicine, Klinikum rechts der Isar, TU Munich, Ismaningerstr. 22 , 81675 Munich, Germany; 2 Department of Trauma and Orthopaedic Surgery, BG Klinikum Unfallkrankenhaus Berlin, Berlin, Germany; 3 Department of Radiology, Klinikum rechts der Isar, TU Munich, Munich, Germany; 4 Gelenkpunkt - Sports- and Joint Surgery Innsbruck, Innsbruck, Austria; 5 UMIT - OSMI- Research Unit for Orthopaedic Sports Medicine and Injury Prevention, Private University UMIT, Hall/ Tirol, Austria

**Keywords:** meniscus repair, Magnetic resonance imaging, meniscus healing, Clinical outcome, Meniscal bucket handle tear

## Abstract

**Background:**

Reports combining patient-reported outcome measures, clinical evaluation, and radiographic assessment of postoperative healing after arthroscopic repair of bucket-handle meniscal tears (BHMT) are scarce.

**Methods:**

Patients who underwent arthroscopic repair for acute traumatic BHMTs between October 2011 and March 2016 with a minimum follow-up of two years were included. Postoperative outcome scores comprised the International Knee Documentation Society Score (IKDC), Lysholm score, Tegner activity score (TAS), and visual analog scale (VAS) for pain. Clinical meniscal healing failure was assessed according to Barrett’s criteria. Side-to-side difference in knee laxity was measured using KT-2000. Radiographic healing was assessed by 3-Tesla magnetic resonance imaging (MRI) and classified according to Henning’s criteria at final follow-up.

**Results:**

Forty patients with a mean age of 32.0 ± 11.5 years were available for follow-up after 51.8 ± 14.3 months. Revision surgery by means of arthroscopic partial meniscectomy was performed in four patients (10%) prior to the follow-up visit. The clinical healing rate was 83.3% at final follow-up. Mean IKDC score was 82.8 ± 13.8 and Lysholm score was 77.4 ± 24.8. Of all patients, 87.5% reached or exceeded the patient-acceptable symptomatic state (PASS) criteria for the IKDC score at final follow-up. The median TAS was 6 and VAS for pain was 0.46 ± 0.9. Side-to-side difference in knee laxity was higher in patients with concomitant ACL reconstruction (2.1 ± 2.7 mm) compared to isolated BHMTs (1.0 ± 2.0 mm). MR examination showed 69.4% healed, 25.0% partially healed, and 5.6% unhealed menisci.

**Conclusion:**

Patients who underwent repair for acute traumatic BHMTs achieved good to excellent clinical outcome along with a high rate of meniscal healing at a minimum follow-up of two years. Clinical and radiological healing rates were similarly satisfactory and most patients exceeded the PASS criteria for the IKDC score. Patients were able to reach a high postoperative activity level.

**Level of evidence:**

Case Series; IV.

## Background

Meniscal tears are one of the most common types of surgically repaired knee injuries [1, 2]. With 10–26% of all meniscal lesions, bucket-handle meniscal tears (BHMT) present a challenging subgroup of these injuries, typically involving a vertical or oblique longitudinal tear with displacement into the intercondylar notch or around the fermoral condyle [3–6]. Thus, clinical symptoms usually include mechanical locking, pain, and perceived instability [6–8].

As the menisci are critical for load transmission, stabilization, lubrication, and proprioception of the knee joint, preservation of meniscal tissue is of great clinical importance for maintaining sufficient function [7–10]. Although partial meniscectomy may reduce pain along with functional improvement in the short-term, removal of meniscal tissue results in cartilage degeneration, premature development of osteoarthritis, and poor long-term outcomes [7, 9, 11–14]. Consequently, BHMTs should undergo repair whenever possible to avoid loss of meniscus volume, with various repair techniques being proposed in current literature mainly determined by the surgeon’s preference [1, 2, 7, 9].

Clinical reports following repair of BHMTs demonstrated satisfying functional outcomes along with a failure rate varying between 10.4 and 34.2% of cases [1, 7, 8, 11, 15–18]. Several factors influencing healing after repair have been identified, including patient age and sex, activity level, anterior cruciate ligament (ACL) laxity, concomitant ACL reconstruction, tear length and chronicity, and rim width [8, 17]. While most of these factors only seem to have negligible impact on postoperative success, concomitant ACL reconstruction has been shown to result in a lower risk of failure when compared to isolated BHMT repairs [8, 15].

However, studies combining patient-reported outcome measures, clinical evaluation, and radiographic assessment of postoperative healing are very limited [15, 19–21]. Thus, the purpose of the present study was to evaluate clinical and radiological outcomes of patients undergoing repair for acute bucket-handle meniscal tears as well as to assess the postoperative healing rate. It was hypothesized that patients who underwent repair for acute BHMTs would demonstrate good to excellent outcome along with high postoperative healing potential at a minimum follow-up of two years.

## Methods

This study was conducted to investigate the clinical outcome and the healing rate of BHMTs in the mid-term follow up. The study was approved by the Institutional Review Board (No.: 307/16) and conducted according to the Declaration of Helsinki. All patients gave their written informed consent to participate.

A chart review of medical records was performed and 56 patients with acute traumatic BHMT were identified and contacted for this study. Inclusion criteria comprised arthroscopic repair of an acute BHMT in patients from epiphyseal closure to 60 years after a minimum follow-up of 24 months. Patients with a concomitant primary ACL rupture were also included. Exclusion criteria contained degenerative meniscus lesions without reported trauma and meniscus root tears. Patients with previous knee surgery, chondral lesions, osteoarthritis > grade II according to Kellgren/Lawrence and multi-ligament injuries were also excluded.

### Clinical assessment

All patients were clinically assessed at final follow including manual meniscus and knee stability testing (Lachman and Pivot-Shift Test) as well as the documentation of range of motion (ROM) by the use of a goniometer. The clinical examination also included a KT-2000 arthrometer measurements (MEDmetric, San Diego, CA, USA) by a knee-trained orthopedic surgeon (LNM) at final follow-up. The KT-2000 arthrometer measurements were performed to assess anterior tibial translation measured in millimeters using a standardized 134 N anterior drawer force at 30° knee flexion and side-to-side differences were recorded.

Clinical meniscal healing failure was defined according to Barrett’s criteria including the presence of either (1) swelling, (2) clicking or blocking, (3) tenderness of joint line, and (4) a positive McMurray test [22]. Presence of one point defined Barrett’s criteria as clinical failure. Objective and subjective clinical outcome scores were obtained via the International Knee Documentation Society Score (IKDC) and Lysholm Score to assess the subjective knee function. Tegner activity score (TAS) and visual analog scale (VAS) were used to measure postoperative sports level and pain level, respectively. Patients’ satisfaction with the treatment was score from 0 (not at all) to 10 (completely satisfied).

The patient-acceptable symptomatic state (PASS) threshold was employed as a tool to assess the minimum score associated with patient satisfaction [23]. In meniscal repair populations, a final IKDC score of 69.0 has been reported to correspond with the PASS [24].

### Radiological assessment

**Fig. 1:**
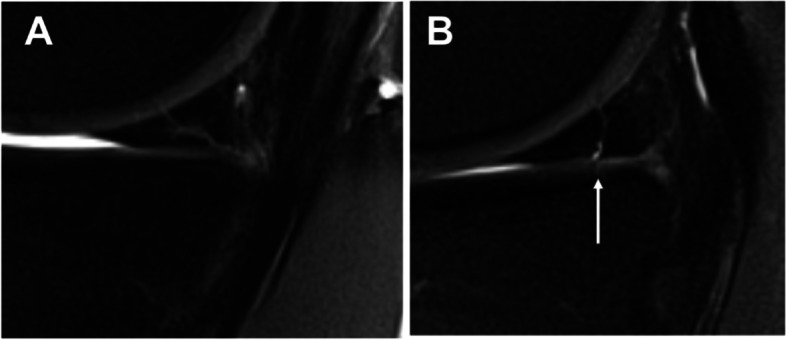
Sagittal fat-suppressed T2-weighted MR images of the knee show (**A**) a healed medial meniscus with no intrameniscal fluid (Henning type I) and **B**) a partially healed meniscus with fluid-equivalent signal in less than 50% of the meniscal height (corresponding to Henning type 2)

Postoperative MR examinations were performed at final follow-up on a 3 Tesla whole-body MR scanner with use of a dedicated 8-channel knee coil (Ingenia, Philips, Best, The Netherlands). The following pulse sequences were acquired with a section thickness of 3 mm: coronal and sagittal T1-weighted turbo spin echo (TSE) sequences with a driven equilibrium (DRIVE) pulse for native arthrographic contrast as well as coronal and sagittal intermediate- and T2 weighted TSE sequences with spectral fat saturation meniscus healing was classified according to Henning’s criteria in (1) healing, (2) partial healing, and (3) non-healing [25]. The meniscus was classified as non-healed if fluid-equivalent signal was present in the tear zone in more than 50% of the tear size (Fig. 1). Two orthopedic knee surgeons (LW and LNM) rated MR images and consensus was obtained in case of primary disagreement for final analysis.

### Surgical technique

All patients underwent arthroscopic surgery for BHMT in one department by experienced orthopaedic knee surgeons as also previously described [26]. A tourniquet was used at 280mmHg and Cefuroxim 1.5 g was administered as perioperative prophylaxis. A routine diagnostic assessment of the intraarticular structures was performed with a 30° arthroscope. The torn meniscus was repaired using either all-inside (AI, Fast-Fix, Smith & Nephew, Andover, MA, USA) or sutures in inside-out (IO) technique depending on tear location. Sutures were placed every 5 mm to provide reliable repair strength. Non-absorbable Fiberwire sutures (Arthrex Inc, Naples, Florida, USA) were used for inside-out technique. In case of concomitant ACL rupture a reconstruction with ipsilateral semitendinosus tendon was performed. A suspension bridge fixation (TightRope®, Arthrex Inc, Naples, Florida, USA) was used femoral fixation and an interference screw tibially.

For postoperative management, all patients had their operated leg secured in a brace for 6 weeks. ROM was restricted to either 90° or 60° of flexion after medial meniscus and lateral meniscus repair, respectively. In the case of medial meniscus repair, weight bearing was only allowed in full extension, after lateral meniscus repair weight bearing was prohibited for 6 weeks. Patients received physiotherapy at a minimum of two times a week.

### Statistical analysis

Descriptive statistics are presented as mean ± standard deviation (SD) for all continuous variables. Frequencies (n, %) were used to obtain descriptive statistics for all categorical variables. Fisher’s exact test was used to analyze for any association between meniscus integrity and demographic variables and injury/surgical factors. Student’s t- test was used to calculate group differences of continuous variables. Interrater correlation coefficient (ICC) was calculated for inter-rater reliability. Statistical significance was set at a *p* value of < 0.05. Data was analyzed using SPSS statistics software version 23.0 (IBM, New York, USA).

## Results

Forty patients with a mean age of 32.0 ± 11.5 (range, 15–58 years) who underwent BHMT repair between October 2011 and March 2016 were available for follow up after 51.8 ± 14.3 months. Patients’ characteristics and surgical data are summarized in Table 1. Revision surgery by means of arthroscopic partial meniscectomy was performed in four patients (10%) prior to the follow-up visit. These patients were included for failure analysis but excluded from clinical outcome score evaluation and radiological assessment due to previous failure of treatment.

**Table 1:** Patients’ characteristics and intraoperative data

Age (years)		32.0 ± 11.5
BMI (kg/m^2^)		25.4 ± 3.5
Time from injury to surgery (d)		12.3 ± 12.1
Sex	Male	27 (67.5%)
	Female	13 (32.5%)
Side	Right	29 (72.5%)
	Left	11 (27.5%)
Laterality	Medial	24 (60%)
	Lateral	16 (40%)
Surgery	ACL reconstruction	26 (65%)
	Isolated meniscus repair	14 (35%)

*Abbreviations*: *BMI* Body mass index, *ACL* Anterior cruciate ligament

### Clinical outcome

The clinical healing rate according to Barrett’s criteria was 83.3% at final follow-up. Three patients reported persistent swelling, 3 had an occasional locking phenomenon, and one had joint line tenderness. Mean subjective IKDC score was 82.8 ± 13.8 and Lysholm score was 77.4 ± 24.8. Of all patients, 87.5% reached or exceeded the PASS criteria for the IKDC score at final follow-up. The median of the Tegner activity scale was 6 (range, 2–9). Patients’ satisfaction was 8.8 ± 1.8 out of 10 and pain level measured by VAS was 0.46 ± 0.9. Knee laxity compared to the healthy contralateral knee measured using KT-2000 was higher in combined ACL and meniscal injuries (2.1 ± 2.7 mm) than in isolated BHMT (1.0 ± 2.0 mm).

Age, sex and concomitant ACL reconstruction had no influence on the clinical healing rate or clinical outcome scores at final follow up (*P* > 0.05, respectively).

### Radiological outcome

Postoperative MR examination at final follow-up showed 69.4% healed, 25.0% partially healed, and 5.6% unhealed menisci. Interrater reliability (ICC) between the two readers was 0.87 (95% CI, 0.75–0.93) and therefore, considered excellent. Both radiologically unhealed menisci were lateral, and one of these patients reported swelling of the knee. Clinical and radiological meniscus healing was not statistically correlated. Patients’ specific risk and intraoperative factors were not correlated with healing seen on MRI at final follow-up.

### Risk factor analysis

Four patients who underwent APM prior to follow up and two patients with an unhealed meniscus on MRI were classified as healing failures. Associations between meniscal healing and demographic characteristics as well as injury/operative data are shown in Table 2.

**Table 2:** Patients’ characteristics and intraoperative data in relation to the meniscal healing failure

		**Bucket handle meniscal tear**		Odds ratio	*P* - value
		**healed**	**failure**		
Sex	Male	21 (77.8%)	6 (22.2%)		*p* = 0.152
	Female	13 (100%)	0 (0%)		
Age	< 30 years	19 (95%)	1 (5%)	OR = 6.3	*p* = 0.182
	>= 30 years	15 (75.0%)	5 (25.0%)		
BMI	BMI < 25	17 (94.4%)	1 (5.6%)	OR = 5.0	*p* = 0.197
	BMI > 25	17 (77.3%)	5 (22.7%)		
Laterality	medial	20 (83.3%)	4 (16.7%)	OR = 1.4	*p* > 0.999
	lateral	14 (87.5%)	2 (12.5%)		
ACL	reconstruction	22 (84.6%)	4 (15.4%)	OR = 1.1	*p* > 0.999
	intact	12 (85.7%)	2 (14.3%)		
Smoking	no	29 (85.3%)	5 (14.7%)	OR = 1.2	*p* > 0.999
	yes	5 (83.3%)	1 (16.7%)		
Time from Injury to Surgery	< 14 days	23 (92.0%)	2 (8.0%)	OR = 4.3	*p* = 0.154
	>= 14 days	8 (72.2%)	3 (27.3%)		

Data is given as numbers (percentage)

*Abbreviations*: *BMI* Body mass index, *ACL* Anterior cruciate ligament

## Discussion

The most important finding of the study was that patients who underwent repair for acute BHMTs achieved good to excellent clinical outcomes along with postoperative healing at a minimum follow-up of two years postoperatively. More specifically, the clinical healing rate according to Barrett’s criteria was 83.3% at final follow-up, while the postoperative MR examination demonstrated 69.4% completely and 25.0% partially healed menisci following BHMT repair, respectively. Of all patients, 87.5% reached or exceeded the PASS criteria for the IKDC score at final follow-up. Further, no correlations were revealed between meniscal healing and demographic characteristics as well as injury patterns. These findings are consistent with previous studies reporting satisfying functional outcomes along with a failure rate ranging between 10.4 and 34.2% of cases [1, 7, 8, 11, 15–19, 21, 27].

BHMTs have been found to account for 10–26% of all meniscal lesions, thus presenting a challenging subgroup of these injuries [3–6]. As preservation of meniscal tissue is of great clinical importance for maintaining sufficient function as well as preventing cartilage degeneration and the premature development of osteoarthritis, BHMTs should undergo surgical repair whenever possible [7–14]. When evaluating clinical outcomes of 38 patients who underwent BMHT repair, Hupperich et al. reported a mean Lysholm score of 86.6 ± 13.5, IKDC of 86.5 ± 10.2, and Tegner activity scale of 6.2 ± 2.2 after an average of 44.4 months postoperatively [7]. However, the authors also found a relatively high clinical failure rate, defined as meniscus re-tear, of 34.2% of patients, subsequently undergoing revision surgery [7]. In contrast, the present study demonstrated a revision rate of 10% of patients who consequently underwent arthroscopic partial meniscectomy, while similar postoperative values in terms of patient-reported outcome scores were achieved. Further, a satisfactory clinical healing rate of 83.3% according to Barrett’s criteria was observed after a mean follow-up of 51.8 months, which is consistent with previous work by Feng et al.[17] and Espejo-Reina et al.[15], reporting clinical healing in 86.6% after 26 months and 83.0% after 48.0 months, respectively.

Although second-look arthroscopy has been reported to most accurately determine meniscal healing, MRI has also been found to demonstrate a high specificity in detecting healed meniscal repairs without need for additional surgery, emphasizing its use as a significant complement to the clinical assessment [28]. However, compared to the clinical healing rate of 83.3% of cases in the present study, the postoperative MR evaluation revealed a slightly lower rate of 69.4% completely healed menisci at final follow-up, while 25.0% were only partially healed and 5.6% were unhealed. Studies evaluating radiological healing following repair of acute BHMTs are scarce. In the setting of chronic medial BHMTs, Espejo-Reina et al. observed a comparable radiographic healing rate of 70.8% at 48 months postoperatively [15]. However, 29.2% of repaired menisci were unhealed at final follow-up, which may be due to large time interval between injury and surgery with a mean of 10 months and the patients’ high activity level [15]. Uzun et al. showed that 5 of 43 (11.7%) all-inside repairs of the lateral meniscus failed after a mean period of 12.8 months postoperatively on MRI [21]. Similarly, Goh et al. showed that 19 of 21 patients had a stable reduction after repair of BHMT on MRI 2 years postoperatively [19].

Failure rates following repair of BHMTs have been reported to vary between 10.4 and 34.2% of cases [1, 7, 8, 11, 15–18, 29], which is consistent with the overall failure rate of 15.0% observed in the present study, comprising four patients who had to undergo revision surgery due to re-tears and two patients who revealed radiographically unhealed meniscal repairs at final follow-up. As the probability for absence of failure has been shown to constantly decrease over time [16], the present findings can be considered as satisfactory results. More specifically, Ardizzone et al. recently found a significantly higher failure rate following BHMT repair in patients with a follow-up period of more than 30 months (34.4%) when compared to patients with a follow-up of less than 30 months postoperatively (23.4%) [1].

Several factors having an impact on failure rates after BHMT repair have been suggested, including patient age and sex, activity level, ACL laxity, concomitant ACL reconstruction, meniscus laterality, delayed surgery, and length of postoperative follow-up [1, 7, 8, 15–18]. Previous studies highlighted the correlation of male sex with significantly increased failure rates following BHMT repair when compared to female patients [1, 7]. Similarly, all of the six failures in the present study occurred in male patients, however, this finding did not reach statistical significance mainly due to the limited sample size, introducing the concern over a type II error. Further, meniscus laterality did not seem to influence postoperative healing with the medial (16.7%) and lateral (12.5%) meniscus showing similar failure rates, which is consistent with recent work [1]. Smoking was identified as risk factor for meniscus healing in one study [21]. Time from injury to surgery has no statistically significant effect in the presented cohort.

While most of these factors rather seem to have negligible impact on postoperative success, several previous studies found concomitant ACL reconstruction to result in a lower risk of failure when compared to isolated BHMT repairs [7, 8, 15]. Espejo-Reina et al. reported that patients with isolated BHMT repairs were 21.3 times more likely to fail compared to those who underwent concomitant ACL reconstruction [15]. However, more recently published studies found that concurrent ACL reconstruction only trended toward being a factor associated with successful repair, without reaching statistical significance [1, 8, 11]. Similarly, the present study showed that concomitant ACL reconstruction was not correlated to meniscal healing. Further, a mean side-to-side difference in knee laxity of 2.1 mm was observed in the ACL reconstruction group, which is consistent with pervious results of Feng et al. with a mean difference of 1.8 mm compared to the healthy contralateral side [17].

As a clinical consequence, acute and traumatic BHMTs should be repaired, and meniscal tissue preserved whenever possible. Clinical and radiographic outcomes demonstrate good to excellent results with a low revision and non-healing rate along with high patient satisfaction.

There were several limitations to the study. Although data was collected prospectively, the chart review was performed retrospectively, potentially creating selection bias. Second, the sample size was limited because only patients with complete data collection comprising patient-reported outcome scores, clinical examination, and MR examination were included in the final analysis. In addition, with reporting outcomes of only a single institution’s practice, external validity may be limited in terms of both patient population and surgical technique. Lastly, sensitivity and specificity of native MR examination is limited regarding the postoperative assessment of meniscal healing.

## Conclusion

Patients who underwent repair for acute traumatic BHMTs achieved good to excellent clinical outcomes along with postoperative healing at a minimum follow-up of two years. Clinical and radiological healing rates were similarly satisfactory and most patients exceeded the PASS criteria for the IKDC score. Patients were able to reach a high postoperative activity level.

## Data Availability

The datasets used and/or analysed during the current study available from the corresponding author on reasonable request.

## Abbreviations

ACL: Anterior cruciate ligament
BHMT: Bucket-handle meniscal tear
ICC: Interrater correlation coefficient
IKDC: International Knee Documentation Society Score
PASS: Patient-acceptable symptomatic state
ROM: Range of motion
SD: Standard deviation
TAS: Tegner activity score
TSE: Turbo spin echo
VAS: Visual analog scale
